# Effects of internet-based supportive intervention on knowledge, pregnancy stress, fear of childbirth, and pregnancy outcomes in primiparas

**DOI:** 10.3389/fmed.2026.1795397

**Published:** 2026-07-09

**Authors:** Fangqin Tu, Yunping He, Jingjie Wang, Yujia Qian

**Affiliations:** Department of Obstetrics, Maternal and Child Health Hospital of Linping District, Hangzhou, Zhejiang, China

**Keywords:** antenatal education, fear, infant, internet-based intervention, pregnancy outcomes, primiparity, stress

## Abstract

**Background:**

The proliferation of internet-based technologies has enabled the development of supportive intervention that have proven efficacious in several health education fields. However, their specific utility for primiparas has yet to be systematically evaluated.

**Methods:**

A total of 332 primiparas were enrolled between January 2021 and December 2023 to participate in a randomized controlled trial, and were equally assigned to two groups (166 per group). The control group received conventional health education, while the intervention group received an internet-based supportive intervention. After excluding dropouts, 141 participants in the intervention group and 137 in the control group were included for per-protocol analysis. Maternal and neonatal health knowledge, pregnancy stress, and fear of childbirth (FOC) were evaluated using the Maternal and Infant Knowledge Scale, Pregnancy Stress Rating Scale (PSRS), and Childbirth Fear Questionnaire (CAQ), respectively. Maternal and neonatal clinical outcomes were also recorded.

**Results:**

After the intervention, the intervention group demonstrated significantly higher knowledge scores in nine out of ten domains, with a higher post-intervention total score (78.45 ± 5.97 vs. 67.31 ± 7.04, *P* < 0.001). Similarly, the intervention group reported significantly lower pregnancy stress across all four domains, with a lower post-intervention total score (31.67 ± 6.85 vs. 38.92 ± 8.76, *P* < 0.001). FOC was also significantly lower in the intervention group across three dimensions, with a lower post-intervention total score (23.56 ± 4.73 vs. 28.24 ± 5.81, *P* < 0.001). Furthermore, sensitivity analyses under the intention-to-treat (ITT) principle showed that these findings were robust to missing data, confirming the benefits of the intervention group. The intervention group had a significantly higher rate of spontaneous vaginal delivery and lower incidences of gestational diabetes, macrosomia, low birth weight, as well as lower overall incidences of adverse maternal and neonatal events (21.28% vs. 35.04%, *P* = 0.011; 19.86% vs. 34.31%, *P* = 0.007, respectively). However, sensitivity analyses showed that adverse events were not robust to missing data, and therefore these results should be interpreted with caution.

**Conclusion:**

The internet-based supportive intervention is associated with higher knowledge, lower stress and FOC, and potential maternal and neonatal benefits among primiparas, suggesting its value as an antenatal education strategy.

## 1 Introduction

Motherhood transition poses multiple challenges, requiring women to acquire parenting skills, adapt to changing family relationships, and embrace their maternal role (1). Primiparas face particular difficulties due to their lack of experience, often exhibiting limited knowledge of pregnancy management, delivery skills, breastfeeding techniques, and other aspects of perinatal care (2). This knowledge deficit renders them more susceptible to negative emotions and maladaptive coping styles compared to multiparas, potentially compromising maternal-child health (3). Many first-time mothers struggle with these adjustments, frequently encountering parenting difficulties and poor role transition that adversely affect both maternal well-being and infant development. Although evidence indicates that maternal health education can equip women with essential knowledge and enhance self-efficacy (4), conventional intervention typically positions pregnant women as passive recipients of standardized information delivered through routine prenatal classes. While valuable, this model consumes considerable healthcare resources and is constrained by environmental and individual factors, leading to variabilities in educational effectiveness (5). These limitations underscore the need for a more flexible, accessible, and individualized antenatal education intervention.

The internet and social media have emerged as important sources of information for pregnant women, giving rise to an internet-based supportive intervention (also termed online health education, e-health, or web-based intervention) that leverages modern media to deliver comprehensive and diversified health education, thereby enhancing acceptance and engagement (6, 7). These interventions draw on cognitive-behavioral principles and align with the behavioral logic of the Knowledge-Attitude-Practice model (8, 9). As environmental, technological, and demographic contexts evolve, such educational models must be continuously adapted to maximize health benefits while remaining feasible and user-friendly. Although this intervention has been increasingly studied in maternal cohorts, with demonstrated benefits in improving knowledge acquisition, alleviating anxiety and depression, and enhancing clinical outcomes, previous studies have primarily focused on general psychological issues and outcomes (10–12). Evidence regarding its effects on pregnancy stress and fear of childbirth (FOC), a distinct psychological challenge, remains insufficient. Furthermore, comprehensive evaluations integrating health knowledge, pregnancy stress, FOC, and clinical outcomes, particularly among primiparas, are scarce. A more nuanced understanding of this specific subgroup is therefore necessary to address this research gap.

This study aimed to test whether the benefits of an internet-based supportive intervention extend to maternal health education. Specifically, we hypothesized that this approach would provide a stronger foundation than conventional education, leading to more favorable outcomes in health knowledge, pregnancy stress, and FOC, as well as maternal and neonatal outcomes. For this purpose, a comparative analysis was designed to evaluate its advantages, limitations, and feasibility.

## 2 Participants and methods

### 2.1 Participants

From July 2021 to December 2023, a total of 332 eligible primiparas provided informed consent and were enrolled through posters or outpatient visits. This study was an open-label, randomized controlled trial because participants and intervention providers could not be blinded due to the nature of the intervention. The inclusion criteria were as follows: (1) singleton pregnancy conceived naturally; (2) normal external pelvic measurements; (3) no cognitive, communication, or reading impairments; and (4) proficiency in using the Internet, WeChat, and other social media tools. The exclusion criteria were: (1) aged <18 or >34 years; (2) gestational age >13 weeks at enrollment; (3) impaired hepatic, renal, coagulation function, or other severe comorbidities; and (4) conditions such as AIDS, hemolytic disease of the fetus, or recurrent spontaneous abortion. Sample size was calculated using G*Power (version 3.1). Based on a two-sided *z*-test for two independent proportions, with a significance level of α = 0.05, power of 80%, and expected incidence rates of 18% in the intervention group and 34% in the control group (derived from our pilot study of 50 cases per group), the required sample size was 117 participants per group. Assuming a 20% dropout rate, the target sample size was set at 147 participants per group. By December 2023, 166 participants had been enrolled in each group, exceeding the required sample size. Eligible participants were randomly assigned in a 1:1 ratio to either the intervention group or the control group (166 per group) using a random sequence generated by an independent researcher and sequentially numbered, opaque, sealed envelopes. After enrollment, the next envelope was opened in order, and the participant was assigned to the group indicated inside, ensuring allocation concealment. To minimize contamination between groups, the internet-based supportive intervention was delivered through personal online platforms and separate WeChat groups, with participants in the two groups having no prior acquaintance. At 1-month postpartum follow-up, 141 participants in the intervention group and 137 in the control group had completed the study, resulting in 25 and 29 cases of attrition, respectively. In the intervention group, attrition was due to requested withdrawal (9 cases), refusal to complete surveys (7 cases, defined as non-completion after three reminders), loss to follow-up (6 cases), and failure to complete intervention protocol (3 cases, defined as not achieving a passing knowledge score of ≥60 after three attempts). Correspondingly, in the control group, 12, 8, 7, and 2 cases were attributed to the same reasons, respectively. In the primary analysis, participants who experienced attrition were excluded, and only completers of the intervention and follow-up were included for per-protocol analysis.

### 2.2 Methods

The control group received conventional health education, including maternal examinations, fetal assessment, and regular follow-up. Upon initial health record establishment, participants received a health education manual. After admission, participants were oriented to hospital protocols, and subsequent maternal and neonatal care followed standard clinical pathways. For the intervention group, we established a multi-component internet-based supportive intervention program, explicitly framed as a complex intervention. Its structure and key elements are described below (13–15). (1) Who provided? The team consisted of one principal leader, three deputy leaders, several staff members, and senior consultants. All leadership roles were held by healthcare professionals with at least 10 years of clinical experience in obstetrics. (2) How was it delivered? The intervention was delivered entirely online via WeChat groups and social media platforms (e.g., Little Red Book, Douyin, WeChat Official Accounts). Participants were clustered by gestational stage (within a 3-month window) into WeChat groups of several dozen members. The research team provided the overall framework, core content, and guidance. Each group also included two trained research staff who released materials or links 2–3 times per week and addressed daily questions. (3) How did participants engage? The intervention took place remotely via internet-connected devices (smartphones, tablets, or computers) in participants’ homes or any location of their choice, allowing them to access materials at their own pace. Participants could read text, listen to audio, or watch videos during free time. The platforms also provided personalized content recommendations based on viewing history. (4) How long did it last? The intervention lasted 6–9 months. It began with a 3-month structured phase covering knowledge from pre-pregnancy to newborn care, including all trimesters, delivery, and postpartum recovery, followed by 3–6 months of stage-specific review and reinforcement. (5) What materials were used? The intervention included three types of multimedia materials: (i) text-based materials (approximately 100 original short articles, each about 1000 words, readable in about 10 h); (ii) audio-based materials (approximately 10 h total, each segment lasting 5–30 min); and (iii) video-based materials (approximately 15 h total, including short videos of 30 s–5 min and long videos of 15–30 min). The core information was consistent across all formats, with variations mainly in presentation style and depth. (6) How was content quality and comprehensiveness ensured? Materials were developed based on official government guidelines and protocols detailed in prior studies (13–15), ensuring content quality and comprehensiveness. All materials underwent a review process before release, including initial self- and cross-checks within the team, followed by a final review by an external consultant. Any disagreements were resolved through team discussion. (7) How were adherence and engagement estimated? Adherence was estimated using aggregated backend data from participant-specific accounts, including content activation rate and active time. One month before delivery, participants completed the Maternal and Infant Knowledge Scale to assess learning effectiveness, with a passing score of ≥60 defined as eligible. Participants who failed to achieve a passing score after three attempts were considered as having failed to complete the protocol.

### 2.3 Observational parameters

Several parameters and outcomes were compared between the two groups, including maternal and neonatal health knowledge, pregnancy stress, FOC, and clinical outcomes. The Maternal and Infant Knowledge Scale was used to assess health knowledge based on established instruments (16, 17). This scale comprises 10 domains (e.g., physiological changes, gestational weight management, and pregnancy complications), with 5 items per domain. In this study, each correct response scores 2 points, yielding a maximum total score of 100, and a higher score indicates a greater level of knowledge. The Pregnancy Stress Rating Scale (PSRS) (18) was employed to measure the pregnancy stress of the primiparas. This scale comprises 30 items across four dimensions: stress from parental role identification (15 items), stress from health and safety of self and baby (8 items), stress from bodily changes (4 items), and other stressors (3 items). Items are rated on a 4-point Likert scale (0–3 points), giving a total score range of 0–90. Higher scores reflect greater pregnancy stress. The Childbirth Fear Questionnaire (CAQ) (19) was used to assess FOC among primiparas. This instrument comprises four dimensions: fetal health (5 items), loss of control (4 items), labor pain and injury (4 items), and hospital intervention and environment (3 items), totaling 16 items. Each item is rated using a 4-level Likert scale (1–4 points). The total score range is from 16 to 64 points, with a higher score indicating a greater degree of FOC. All scales were administered at enrollment (before intervention) and at approximately 1 month before delivery (after intervention). The total score of each scale was the primary outcome, while subdomain scores were exploratory. These scales have been previously validated in Chinese pregnant populations, with good reliability and validity. The Maternal and Infant Knowledge Scale showed high internal consistency (Cronbach‘s α = 0.890) and test-retest reliability (*r* = 0.897) (17), and the PSRS also had good internal consistency (Cronbach’s α = 0.920) and test-retest reliability (*r* = 0.820) (18). Furthermore, the CAQ demonstrated good internal consistency (Cronbach’s α = 0.910) and test-retest reliability (*r* = 0.803) (19). In our sample, the three scales also demonstrated good internal consistency, with Cronbach’s α coefficients of 0.856, 0.913, and 0.872, respectively. Clinical outcomes for both mothers and newborns were recorded at 1 month postpartum from hospital medical records and follow-up data. Two researchers independently reviewed the data, with any disagreements adjudicated by an associate chief obstetrician.

### 2.4 Statistical analysis

All statistical analyses were performed using SPSS 25.0. To minimize detection bias, data collection and verification were performed by research team assessors blinded to group assignment, while statistical analyses were performed by an independent statistician also blinded to group allocation. Non-normally distributed continuous data are presented as median (interquartile range, IQR), whereas normally distributed data are presented as mean ± standard deviation (SD). Intergroup differences were assessed using independent-samples *t*-test, and within-group changes from baseline were evaluated using paired-samples *t*-test. Categorical data were summarized as frequencies or percentages (%), and intergroup comparisons were conducted using the Chi-square (χ^2^) test or Fisher’s exact test. Ranked data were analyzed by the rank-sum test. Results were reported as mean difference (95% CI) for continuous variables and odds ratios (OR) with 95% CI for categorical variables. A two-sided *P* < 0.05 was considered statistically significant. To assess the robustness to attrition, a worst-case sensitivity analysis was performed following the intention-to-treat (ITT) principle. For continuous outcomes (total knowledge, PSRS, and CAQ scores), missing post-intervention values in the intervention group were replaced by the baseline mean (assuming no improvement). For the control group, missing values were imputed using favorable outcome values: the 90th percentile of completers’ scores for total knowledge (higher = better), and the 10th percentile for total PSRS and CAQ scores (lower = better). For binary clinical outcomes, all dropouts in the intervention group were assumed to have the event, and none in the control group. Robustness was inferred if the direction and significance remained unchanged under these extreme assumptions.

## 3 Results

### 3.1 Comparison of general characteristics

There were no significant differences between the two groups in general characteristics, including age, residence area, education status, occupations, marital status, pregnancy intention, and family income (*P* > 0.05), indicating that the groups were comparable. The detailed data were presented in Table 1.

**TABLE 1:** Comparison of general characteristics in the two groups.

Characteristics	Intervention group (*n* = 141)	Control group (*n* = 137)	χ ^2^ / Hc	*P*
Age (years)			2.803	0.246
18–25	34 (24.11)	40 (29.20)		
26–30	83 (58.87)	67 (48.91)		
31–34	24 (17.02)	30 (21.90)		
Residence area			0.648	0.421
Urban	92 (65.25)	83 (60.58)		
Rural	49 (34.75)	54 (39.42)		
Education status			1.343	0.511
High school or equivalent	43 (30.50)	38 (27.74)		
Bachelor’s degree	72 (51.06)	66 (48.18)		
Master’s degree or higher	26 (18.44)	33 (24.09)		
Occupations			2.018	0.364
Employed	83 (58.87)	90 (65.69)		
Household manager	30 (21.28)	28 (20.44)		
Self-employed	28 (19.86)	19 (13.87)		
Marital status			0.457	0.499
Married	136 (96.45)	134 (97.81)		
Single/others	5 (3.55)	3 (2.19)		
Pregnancy intention			1.232	0.267
Planned	110 (78.01)	99 (72.26)		
Unplanned	31 (21.99)	38 (27.74)		
Family income (CNY/month)			0.792	0.673
<10,000	20 (14.18)	23 (16.79)		
10,000–20,000	72 (51.06)	63 (45.99)		
>20,000	49 (34.75)	51 (37.23)		

### 3.2 Comparison of knowledge

Baseline knowledge domains did not differ significantly between groups (*P* > 0.05). After the intervention, both groups exhibited significant within-group improvements in each domain and total score. In the intervention group, the median content activation rate was 65.7% (IQR: 31.2%–87.5%), and the median total active learning time was 28.1 h (IQR: 15.8–51.2 h). For intergroup comparison, the post-intervention domain and total scores of the intervention group were significantly superior to those of the control group (*P* < 0.05), with the exception of gestational weight management (*P* > 0.05). The results were shown in Table 2. Under the worst-case imputation, ITT sensitivity analysis confirmed that the intervention group maintained a significantly higher total knowledge score (ITT mean: 74.38 vs. 69.13; mean difference = 5.25, 95% CI: 3.64–6.86, *P* < 0.001), indicating that the finding is robust to missing data.

**TABLE 2:** Comparison of knowledge in the two groups.

Domains	Intervention group (*n* = 141)	Control group (*n* = 137)	Mean difference (95% CI)	*P*
Physiological changes				
Before intervention	4.87 ± 1.58	5.15 ± 1.82	–	0.172
After intervention	7.42 ± 2.11*	6.79 ± 1.78*	0.63 (0.18–1.08)	0.008
Gestational weight management				
Before intervention	4.16 ± 1.45	3.86 ± 1.38	–	0.078
After intervention	7.14 ± 1.94*	6.71 ± 2.25*	0.43 (−0.06 to 0.92)	0.089
Nutrition and dietary management				
Before intervention	5.63 ± 2.08	5.45 ± 1.72	–	0.4338
After intervention	8.38 ± 1.25*	7.22 ± 1.58*	1.16 (0.82–1.50)	<0.001
Pregnancy complications				
Before intervention	2.80 ± 1.18	3.04 ± 1.30	−1.1032 to −0.1368	0.108
After intervention	5.51 ± 2.17*	4.83 ± 1.91*	0.68 (0.20–1.16)	0.006
Preparation before delivery				
Before intervention	3.77 ± 1.86	4.08 ± 2.12	–	0.219
After intervention	8.37 ± 1.25*	7.73 ± 1.47*	0.64 (0.33–0.95)	<0.001
Skills of delivery				
Before intervention	4.94 ± 1.50	5.23 ± 1.45	–	0.103
After intervention	9.12 ± 0.64*	6.76 ± 2.32*	2.36 (1.96–2.76)	<0.001
Health care during puerperium				
Before intervention	5.03 ± 1.83	4.97 ± 1.69	–	0.777
After intervention	7.95 ± 1.36*	7.19 ± 1.53*	0.76 (0.43–1.09)	<0.001
Post-birth recovery				
Before intervention	5.86 ± 2.30	6.04 ± 2.12	–	0.498
After intervention	9.01 ± 0.80*	7.15 ± 1.44*	1.86 (1.58–2.14)	<0.001
Neonatal feeding and care				
Before intervention	6.48 ± 1.89	6.13 ± 2.25	–	0.161
After intervention	8.42 ± 1.12*	7.08 ± 1.63*	1.34 (1.01–1.67)	<0.001
Common neonatal diseases				
Before intervention	4.11 ± 1.53	3.92 ± 1.45	–	0.289
After intervention	7.15 ± 1.74*	5.86 ± 1.50*	1.29 (0.91–1.67)	<0.001
Total score				
Before intervention	47.67 ± 6.52	47.88 ± 5.61	–	0.774
After intervention	78.45 ± 5.97*	67.31 ± 7.04*	11.14 (9.60–12.68)	<0.001

*Within-group comparison to baseline, *P* < 0.05.

### 3.3 Comparison of pregnancy stress

At baseline, no significant intergroup differences were observed in any domain of pregnancy stress or in the total score (*P* > 0.05). Following the intervention, both groups showed significant within-group reductions in each domain and in the total score (*P* < 0.05). Furthermore, the intervention group demonstrated significantly larger reductions than the control group in all four stress domains and in the total score (*P* < 0.05). The results were shown in Table 3. ITT sensitivity analysis for total PSRS score demonstrated that the intervention group still showed significantly lower total pregnancy stress score than the control group (ITT mean: 34.10 vs. 37.25, mean difference = −3.15, 95% CI: −4.84 to −1.46, *P* < 0.001), confirming the robustness of the intervention effect.

**TABLE 3:** Comparison of pregnancy stress in the two groups.

Domains	Intervention group (*n* = 141)	Control group (*n* = 137)	Mean difference (95% CI)	*P*
Parental role identification				
Before intervention	22.15 ± 7.23	20.87 ± 6.58	–	0.124
After intervention	14.37 ± 5.34*	16.61 ± 7.15*	−2.24 (−3.75 to −0.73)	0.003
Health and safety of self and baby				
Before intervention	15.53 ± 5.18	15.97 ± 5.46	–	0.491
After intervention	10.79 ± 3.75*	14.25 ± 4.32*	−3.46 (−4.43 to −2.49)	<0.001
Bodily changes				
Before intervention	7.14 ± 2.58	6.83 ± 3.17	–	0.371
After intervention	3.68 ± 1.67*	4.82 ± 2.15*	−1.14 (−1.59 to −0.69)	<0.001
Other stressors				
Before intervention	4.95 ± 2.19	5.30 ± 2.43	–	0.208
After intervention	2.83 ± 1.26*	3.24 ± 1.51*	−0.41 (−0.74 to −0.08)	0.014
Total score				
Before intervention	49.78 ± 9.62	48.96 ± 10.13	–	0.489
After intervention	31.67 ± 6.85*	38.92 ± 8.76*	−7.25 (−9.10 to −5.40)	<0.001

*Within-group comparison to baseline, *P* < 0.05.

### 3.4 Comparison of FOC

Prior to the intervention, there were no significant differences between the two groups in any of the four domains or in the total score (*P* > 0.05). After the intervention, while the scores in the domains of fetal health, loss of control, and labor pain and injury, as well as the total score, decreased significantly from baseline in both groups (*P* < 0.05), the intervention group achieved significantly lower scores in these three domains and lower total score compared with the control group (*P* < 0.05). No significant intragroup or intergroup differences were found for the domain of hospital intervention and environment (*P* > 0.05). The results were shown in Table 4. The ITT sensitivity analysis for total CAQ revealed that the intervention group maintained a significantly lower total fear of childbirth score (ITT mean: 25.69 vs. 27.35, mean difference = −1.66, 95% CI: −2.91 to −0.41, *P* = 0.009), supporting the robustness of this finding to missing data.

**TABLE 4:** Comparison of FOC in the two groups.

Domains	Intervention group (*n* = 141)	Control group (*n* = 137)	Mean difference (95% CI)	*P*
Fetal health				
Before intervention	12.48 ± 4.18	13.19 ± 3.65	–	0.133
After intervention	7.43 ± 2.75*	9.25 ± 3.41*	−1.82 (−2.55 to −1.09)	<0.001
Loss of control				
Before intervention	9.59 ± 3.20	10.08 ± 4.04	–	0.263
After intervention	5.73 ± 2.05*	7.81 ± 3.19*	−2.08 (−2.72 to −1.44)	<0.001
Labor pain and injury				
Before intervention	10.25 ± 3.57	9.74 ± 3.22	–	0.212
After intervention	6.60 ± 2.59*	7.53 ± 2.87*	−0.93 (−1.57 to −0.29)	0.005
Hospital intervention and environment				
Before intervention	4.21 ± 2.03	3.99 ± 1.86	–	0.347
After intervention	3.79 ± 1.71	3.63 ± 1.52	0.16 (−0.22 to 0.54)	0.411
Total score				
Before intervention	36.54 ± 7.08	37.02 ± 6.79	–	0.565
After intervention	23.56 ± 4.73*	28.24 ± 5.81*	−4.68 (−5.93 to −3.43)	<0.001

FOC, fear of childbirth, *Within-group comparison to baseline, *P* < 0.05.

### 3.5 Comparison of maternal outcomes

The intervention group had a significantly higher rate of spontaneous vaginal delivery and a significantly lower incidence of gestational diabetes than the control group (*P* < 0.05). The overall incidence of adverse maternal events was 21.28% (30/141) in the intervention group, which was significantly lower than the incidence of 35.04% (48/137) in the control group (*P* < 0.05). Maternal outcomes were presented in Table 5. Under the extreme assumption that all 25 intervention dropouts had the adverse event (or failed to achieve spontaneous vaginal delivery) and none of the 29 control dropouts did, the following ITT estimates were obtained: spontaneous vaginal delivery rate 65.66% (109/166) vs. 68.67% (114/166), OR = 0.87 (95% CI: 0.55–1.38), *P* = 0.559; gestational diabetes incidence 21.08% (35/166) vs. 12.65% (21/166), OR = 1.84 (95% CI: 1.02–3.33), *P* = 0.040; overall maternal adverse events 33.13% (55/166) vs. 28.92% (48/166), OR = 1.21 (95% CI: 0.76–1.94), *P* = 0.406. While spontaneous vaginal delivery and overall adverse events lost statistical significance, gestational diabetes remained significant but reversed in direction. Thus, the protective effects observed in the per-protocol analysis for maternal outcomes are sensitive to missing data, and all findings should be interpreted with caution.

**TABLE 5:** Comparison of maternal outcomes in the two groups.

Maternal outcomes	Intervention group (*n* = 141)	Control group (*n* = 137)	OR (95% CI)	*P*
General maternal parameters				
Mode of delivery			0.50 (0.29–0.86)	0.020
Spontaneous vaginal delivery	109 (77.30)	85 (62.04)*		
Assisted vaginal delivery	11 (7.80)	20 (14.60)		
Cesarean delivery	21 (14.89)	32 (23.36)		
Adverse maternal events				
Gestational diabetes	10 (7.09)	21 (15.33)	0.42 (0.19–0.93)	0.029
Gestational hypertension	5 (3.55)	10 (7.30)	0.47 (0.16–1.41)	0.166
Preeclampsia	3 (2.13)	5 (3.65)	0.58 (0.14–2.47)	0.448
Anemia	4 (2.84)	3 (2.19)	1.30 (0.29–5.92)	0.731
Perineal laceration	4 (2.84)	2 (1.46)	1.96 (0.35–10.87)	0.430
Puerperal infection	2 (1.42)	3 (2.19)	0.64 (0.11–3.90)	0.629
Placental abruption	1 (0.71)	2 (1.46)	0.48 (0.04–5.38)	0.618
Other conditions^△^	1 (0.71)	2 (1.46)	0.48 (0.04–5.38)	0.618
Overall incidence	30 (21.28)	48 (35.04)	0.50 (0.29–0.86)	0.011

^△^ Included postpartum hemorrhage, amniotic fluid embolism, and uterine rupture;

*Compared with the intervention group, *P* < 0.05.

### 3.6 Comparison of neonatal outcomes

There were no significant differences in general neonatal parameters between the two groups (*P* > 0.05). However, the incidence of macrosomia and low birth weight was significantly lower in the intervention group than in the control group (*P* < 0.05). The overall incidence of adverse neonatal events was 19.86% (28/141) in the intervention group, which was significantly lower than the incidence of 34.31% (47/137) in the control group (*P* < 0.05). Neonatal outcomes were presented in Table 6. Under the extreme assumption, ITT estimates were obtained: macrosomia incidence 19.28% (32/166) vs. 10.24% (17/166), OR = 2.09 (95% CI: 1.11–3.94), *P* = 0.020; low birth weight incidence 16.87% (28/166) vs. 6.02% (10/166), OR = 3.17 (95% CI: 1.48–6.75), *P* = 0.003; overall neonatal adverse events 31.93% (53/166) vs. 28.31% (47/166), OR = 1.19 (95% CI: 0.74–1.90), *P* = 0.473. Macrosomia and low birth weight remained significant but favored the control group, while overall neonatal adverse events were no longer significant These findings suggest that the protective effects observed in the per-protocol analysis for neonatal outcomes are not robust to missing data, warranting cautious interpretation.

**TABLE 6:** Comparison of neonatal outcomes in the two groups.

Neonatal outcomes	Intervention group (*n* = 141)	Control group (*n* = 137)	Mean difference / OR (95% CI)	*P*
General neonatal parameters				
Gestational age (weeks)	39.12 ± 1.73	38.76 ± 2.05	0.36 (−0.08 to 0.80)	0.114
Birth weight (g)	3289.48 ± 396.51	3318.60 ± 514.82	−29.12 (−136.58 to 78.34)	0.597
Length (cm)	50.06 ± 1.93	49.87 ± 2.21	0.19 (−0.29 to 0.67)	0.445
Apgar score (points)	8.85 ± 0.86	8.73 ± 1.04	0.12 (−0.10 to 0.34)	0.295
Adverse neonatal events				
Macrosomia	7 (4.96)	17 (12.41)	0.37 (0.15–0.92)	0.027
Low-birth weight	3 (2.13)	11 (8.03)	0.25 (0.07–0.91)	0.024
Fetal distress	3 (2.13)	6 (4.38)	0.48 (0.12–1.95)	0.289
Premature delivery	5 (3.55)	3 (2.19)	1.64 (0.38–7.01)	0.503
Late-term delivery	3 (2.13)	5 (3.65)	0.58 (0.14–2.47)	0.448
Threatened abortion	4 (2.84)	2 (1.46)	1.96 (0.35–10.87)	0.430
Asphyxia	1 (0.71)	2 (1.46)	0.48 (0.04–5.38)	0.618
Other conditions^Δ^	2 (1.42)	1 (0.73)	1.95 (0.17–21.81)	0.627
Overall incidence	28 (19.86)	47 (34.31)	0.47 (0.27–0.81)	0.007

^Δ^ Included intrauterine infection, Neonatal brachial plexus injury, and congenital anomalies.

## 4 Discussion

The internet has become an important resource for health education, and adequate maternal health literacy and health information-seeking behavior are fundamental to alleviating adverse emotions and improving pregnancy outcomes (20, 21). In this study, the intervention group showed greater improvement in health knowledge across most dimensions than the control group. This is likely attributable to the diverse learning resources and formats of the internet-based approach, including text, video, and audio, which strengthened pregnant women’s autonomy and better aligned with different gestational stages (22). A study from Indonesia reported that, compared with traditional interventions, the internet-based approach significantly improved pregnancy knowledge and independence levels (23), suggesting that this approach yields comparable improvements across different regions. Pregnancy stress refers to non-specific stress responses experienced by pregnant women and is highly prevalent, with up to 92.3% of expectant mothers experiencing related symptoms (24). In this study, our results also revealed a significant reduction in pregnancy stress, confirming its efficacy among primiparas. Given that pregnancy stress is a well-established risk factor for maternal mental health and clinical outcomes (25, 26), this reduction may contribute to improved outcomes for both mothers and newborns. However, pregnancy stress is closely associated with social, family, and obstetric support (27). Our study only observed reduced pregnancy stress at the phenotypic level, without investigating underlying drivers or controlling for confounders. Therefore, future mechanism-oriented studies are needed to clarify causal pathways and develop more targeted supportive strategies for pregnant women.

Fear of childbirth is an important yet often neglected maternal health issue that poses a significant psychological challenge during pregnancy and may cause adverse emotional, physical, and psychological consequences, especially in younger pregnant women (28). Previous studies reported a high prevalence of this condition, affecting 56% of primiparas and 23% of multiparas, with rates varying across different countries (29, 30). A study focusing on primiparas’ FOC and self-efficacy found that a tele-midwifery intervention effectively improved self-efficacy and reduced FOC at 8 weeks postpartum and after delivery (31). Assessing FOC at 1 month before delivery, the intervention group similarly disclosed beneficial effects on prenatal FOC in primiparas, and further extended the investigation to pregnancy stress, adding evidence on psychological well-being from a stress perspective. As described by Athinaidou et al. (32), this improvement may be related to enhanced maternal knowledge and skills through antenatal education. Furthermore, Zhang et al. (33) implemented an internet-based supportive intervention for primiparas and reported high acceptance, thereby alleviating FOC and improving breastfeeding confidence. These findings highlight the value of the internet-based supportive intervention in supporting primiparas throughout their maternal journey, benefiting their newborns through improved breastfeeding practices. Notably, no significant intergroup differences were observed regarding fear of hospital intervention and environment. This lack of effect may be related to the relatively fixed nature of the hospital setting or potential biases from a small sample size.

In our per-protocol analysis, the intervention group had significantly higher rates of spontaneous vaginal delivery and lower incidences of gestational diabetes, macrosomia, and low birth weight, as well as fewer overall adverse events. These benefits were supported by a meta-analysis (34), which also noted that high heterogeneity and potential bias warrant further research across different resources and settings. Our study adds evidence from an Asian population in developing countries and reinforces the evidence base for internet-based supportive intervention targeting primiparas. Under this intervention, the approach enhances pregnant women’s nutrition and health literacy, supports lifestyle behavior modification, and helps reduce gestational diabetes and other nutrition-related issues (35). Subsequently, adequate nutrition literacy helps avoid both fetal growth restriction from malnutrition and excessive fetal growth from overnutrition, facilitating a reduction in low birth weight and cesarean deliveries (36). Moreover, a study from Canada indicated that internet-based approach can effectively reduce excessive gestational weight gain and gestational diabetes risk, while also lowering the risks of preeclampsia and preterm birth (37). In our study, we did not observe such benefits for preeclampsia or preterm birth. This discrepancy may be attributed to differences in intervention details across populations with varying ethnic and cultural backgrounds. Overall, these findings highlight the potential of internet-based supportive intervention for primiparas. Nevertheless, unadjusted confounders (including prepregnancy BMI, nutritional status, and baseline health) and insufficient robustness from sensitivity analyses regarding the intervention’s superiority mean our results need to be interpreted cautiously. Future studies should reduce biases and adopt multivariate analyses to clarify causal relationships and confirm inferences.

In the specific population of primiparas, several studies have yielded findings that complement those of the present study. A study from Iran found that internet-based health education improved breastfeeding self-efficacy (38). Another study from Saudi Arabia reported that internet-based approach nearly tripled the rate of exclusive breastfeeding within the first month postpartum, and also increased their intention to continue breastfeeding beyond 6 months (39). Although the present study did not assess breastfeeding, these findings corroborate the advantages of such intervention in promoting breastfeeding. Furthermore, a study from Chile demonstrated that online modalities had high acceptability and practicality, effectively reducing postnatal depression and anxiety among primiparas (40). While these studies differ in their specific focuses, they collectively support the potential of internet-based supportive intervention. Compared with others, our intervention adopted a multi-platform, open-access, guided self-help supportive model that is compatible with various digital devices and apps, and places greater emphasis on flexibility and individual preferences. However, this model may be associated with suboptimal adherence among individuals with limited self-learning ability or low self-discipline. Therefore, researchers from Denmark (41) and Iran (42) have proposed the use of mHealth or an “all-in-one” solution to achieve better usability and adherence. Specifically, cultural, economic, and healthcare system contexts vary across countries, and primiparas may face different risks for negative psychological outcomes and adverse events (43, 44). These contextual differences should be considered when generalizing our findings to other settings, as the intervention’s effect may vary across populations with different backgrounds (45).

This study comprehensively evaluated an internet-based supportive intervention for primiparas, integrating health knowledge, pregnancy stress, FOC, and maternal/neonatal outcomes. To our knowledge, no prior study has combined all these elements, and our findings address this research gap. However, several potential limitations should be acknowledged. First, this single-center study only included individuals proficient with digital tools and social media platforms, introducing selection bias and limiting generalizability to populations with lower digital literacy or limited access to technology. Second, although randomization was employed, we were unable to blind participants to group assignment, as they could easily identify their allocated intervention from the content and format. This may have introduced self-reporting bias, and our findings require further validation in more rigorously designed trials with adequate blinding. Third, the internet-based approach incorporated written, audio, and visual materials with the intention of providing primiparas with diverse options tailored to their preferences. Nevertheless, this complex, multi-component design limits our ability to determine which specific component was most beneficial or to precisely quantify the intervention “dose” for each participant, which constitutes a weakness of the present study. Future dismantling designs are therefore needed to identify its active ingredients. Finally, although dropout rates were balanced, the per-protocol analysis may introduce attrition bias and potentially overestimate the intervention effects. Sensitivity analyses showed that knowledge and psychological results were robust to missing data, whereas clinical outcomes were not under the extreme missing data assumption. Therefore, our adverse event results should be interpreted with caution, and ITT analyses with larger samples are needed to confirm these findings.

In conclusion, the internet-based supportive intervention is associated with higher knowledge, lower stress and FOC, as well as potential maternal and neonatal benefits among primiparas, suggesting that this approach represents a promising antenatal education strategy. However, given the single-center and open-label designs, the results warrant further confirmation through well-designed multicenter randomized controlled trials.

## Data Availability

The raw data supporting the conclusions of this article will be made available by the authors, without undue reservation.
